# Demoralisation and its link with depression, psychological adjustment and suicidality among cancer patients: A network psychometrics approach

**DOI:** 10.1002/cam4.4406

**Published:** 2021-12-04

**Authors:** Irene Bobevski, David W. Kissane, Sigrun Vehling, Anja Mehnert‐Theuerkauf, Martino Belvederi Murri, Luigi Grassi

**Affiliations:** ^1^ Department of Psychiatry School of Clinical Sciences at Monash Health Faculty of Medicine, Nursing and Health Sciences Monash University Clayton VIC Australia; ^2^ Palliative Medicine University of Notre Dame Darlinghurst NSW Australia; ^3^ Department of Medical Psychology University Medical Center Hamburg‐Eppendorf Hamburg Germany; ^4^ Department of Medical Psychology and Medical Sociology University Medical Center Leipzig Leipzig Germany; ^5^ Institute of Psychiatry Department of Biomedical and Specialty Surgical Sciences University of Ferrara Ferrara Italy; ^6^ University Hospital Psychiatry Unit, Integrated Department of Mental Health and Addictive Behavior S. Anna University Hospital and Health Authorities Ferrara Italy

**Keywords:** adjustment disorder, cancer, demoralisation, depression, network analysis, suicidal ideation

## Abstract

**Background:**

Demoralisation is a clinically significant problem among cancer patients with a prevalence of 13%–18%. It is defined by difficulty in adjusting to a stressor, wherein the person feels trapped in their predicament and experiences helplessness, hopelessness, loss of confidence and loss of meaning in life. Demoralisation has a strong link with the desire for hastened death and suicidal ideation among the medically ill. This study explored whether a group of symptoms could be identified, distinct from depression, but consistent with adjustment difficulties with demoralisation and linked to ideation of death and suicide.

**Methods:**

Exploratory Graph Analysis, a network psychometrics technique, was conducted on a large German study of 1529 cancer patients. Demoralisation was measured with the Demoralisation Scale II and depressive symptoms with the PHQ‐9.

**Results:**

A network of symptoms, with four stable communities, was identified: 1. Loss of hope and meaning; 2. Non‐specific emotionality; 3. Entrapment; 4. Depressive symptoms. The first three communities were clearly distinct from the PHQ‐9 depressive symptoms, except for suicidality and fear of failure. Community 1, Loss of hope and meaning, had the strongest association with thoughts of death and suicide. Hopelessness, loss of role in life, tiredness, pointlessness and feeling trapped were the most central symptoms in the network.

**Conclusions:**

Communities 1 to 3 are consistent with poor coping without anhedonia and other classic depression symptoms, but linked to suicidal ideation. For people facing the existential threat of cancer, this may indicate poor psychological adjustment to the stressors of their illness.

## Graphical abstract

Demoralisation is a prevalent and clinically significant problem among cancer patients, more highly associated with suicidal thinking than depression. New psychometric techniques using network analysis provide evidence that demoralisation may be an important element of psychological adjustment, explaining the independent link of adjustment disorder to suicidality and potentially contributing to a more clinically useful conceptualisation of adjustment disorder.

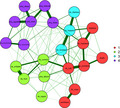

## 1 INTRODUCTION

Demoralisation is a clinically significant problem among cancer patients with a prevalence of 13%–18%,^1^ and higher among other medical illnesses and community settings, 15%–30%.^2^ It is defined by difficulty in adjusting to a stressor, wherein the person feels they are trapped in their predicament and experience feelings of helplessness, hopelessness, loss of confidence and loss of meaning in life.^3^ Demoralisation has a stronger link than depression with the desire for hastened death and suicidal ideation among the medically ill.^4^, ^5^, ^6^, ^7^

Demoralisation is closely related to depression, although evidence shows that it is distinct from anhedonia.^1^ In its most severe form demoralisation is often comorbid with major depressive disorder (MDD).^1^ However, in less severe forms it is more consistent with failure to adapt and cope, characteristic of adjustment disorder (AD). In a recent study,^8^ using latent class analysis (LCA) in a large sample of 1527 German cancer patients, four classes of patients were differentiated: (1) absence of distress; (2) somatic symptoms; (3) severe psychopathology with symptoms of depression, anxiety and demoralisation, including suicidal ideation; and (4) symptoms of demoralisation with suicidal ideation. The fourth class reflected poor coping with suicidal ideation, but no anxiety, nor anhedonia. Class 4 is consistent with the ICD‐11 definition of AD,^9^ according to which poor adjustment to a stressor is the core criteria.

Although AD is considered as a subthreshold disorder, evidence from large studies shows that the rate of suicidal behaviours^10^ and completed suicide^11^ of people with AD is similar to those with MDD. Moreover, compared to MDD, patients with AD become suicidal at lower emotional distress scores, have more stressful life events and higher impulsivity and personality traits associated with suicide.^12^ This is consistent with the results of the above LCA study^8^ which showed suicidal ideation as closely linked to the group with demoralisation/adjustment symptoms, as to the group with more severe psychopathology and depression. AD is among the most frequent of mental disorders among the medically ill, with 11%–19% prevalence.^13^ However it is under‐investigated and under‐recognised^12^, ^13^ due to DSM‐5 and ICD‐10 diagnostic criteria, which are critiqued for being too subjective, lacking specificity and sufficient clinical utility.^12^ Furthermore, evidence for effective interventions for adjustment disorder is lacking, with little support for any specific intervention,^12^ never more so than among the medically ill. Demoralisation may be an important element of adjustment which explains the independent link to suicidality and contributes to a more clinically useful conceptualisation of AD that enables targeting of specific symptoms in therapy.

Previously LCA has been used, which classifies patients into distinct groups using the categorical presence of symptoms.^8^ New techniques, using network analysis,^14^, ^15^, ^16^ allow a more in‐depth examination of the inter‐relationships between dimensional symptoms to understand better how symptoms of demoralisation, poor adjustment and depression are linked. Network theory conceptualises mental disorders as complex networks of causally interconnected symptoms.^14^ Within a graphical representation of the network, symptoms are represented as *nodes*, and the associations between symptoms as *weighted edges* connecting the nodes. Stronger associations (or edge weights) are visually represented by thicker lines. Comorbid disorders have shared nodes. Conversely, the more distinct a group of nodes is from another, the thinner the interconnecting lines will be between them.^17^

Network analysis also provides metrics of *centrality*, indicating where the hub of any community might lie.^18^ The activation of a highly central symptom could potentially generate many related symptoms and cause impairment. Thus, targeting highly central symptoms in intervention and prevention programs before they lead to more severe disorder is of clinical importance.^19^ However, longitudinal studies are needed to establish causality.^20^

A recent development in network psychometrics is exploratory graph analysis (EGA)^17^ which identifies *communities* of nodes closely interconnected with each other but less connected to other nodes in the network. EGA has been used to investigate the structure of mental disorders in a variety of fields, including depression, suicidality and personality.^21^, ^22^

### 1.1 Aims

The present study aimed to use EGA to further examine the phenomenology of demoralisation and depression symptoms in order to obtain a more in‐depth understanding of the inter‐relationships and the relative importance (centrality) of symptoms. We aimed to explore whether this approach is consistent with our previous findings, suggesting the existence of a group of central symptoms, distinct from depression, but consistent with adjustment difficulties with demoralisation, and linked to ideation of death and suicide.

## 2 METHODOLOGY

### 2.1 Sample and procedures

Data from a large German study of 1529 cancer patients were used. The data consisted of a balanced cohort of participants with early stage and advanced cancer from three combined samples. Sample 1 (*n* = 944) was recruited as part of a nationwide epidemiological study representative for all tumour sites and treatment settings in Germany.^23^ Samples 2 (*n* = 270) and 3 (*n* = 315) included adult cancer patients recruited from in‐ and outpatient clinics at a large University Medical Centre. Exclusion criteria were severe physical or cognitive impairment and language problems that interfered with the ability to give informed consent for research. Overall, 1529 out of 2842 eligible patients participated (average participation rate 54%). Approval was received from the institution's Ethics Committee for all studies. The methodology of the study is described in detail elsewhere.^23^

### 2.2 Materials

#### 2.2.1 Demographic characteristics

Demographic characteristics, including age, gender, education level, relationship status and type and stage of cancer were assessed with a standardised self‐report questionnaire.

#### 2.2.2 Demoralization Scale (DS)

Symptoms of demoralisation were measured with the German version of the Demoralisation Scale (DS).^24^ This scale has high internal reliability (α = 0.84) and good construct validity.^24^ The DS has been translated and validated in more than 10 languages, with many studies highlighting its clinical importance across a variety of settings, especially when medical illness is the stressor.^1^, ^2^ More recently, the DS‐II, a refined and abbreviated 16‐item version of the DS, was also developed and validated,^25^ highlighting through item response theory the symptoms of highest clinical importance. Therefore, in the present study we only used the 16 items of the DS‐II.

#### 2.2.3 PHQ‐9

Depressive symptoms were measured with the Patient Health Questionnaire‐9 (PHQ‐9),^26^ which consists of the nine criteria used to diagnose DSM‐IV / 5 depression, each scored on a numeric 0–3 scale. Total scores range from 0 to 27. A cut‐off score of ≥10 is indicative of MDD.

### 2.3 Statistical analysis

All analysis was conducted with R 4.0.0.^27^

#### 2.3.1 Internal reliability

Internal reliability of the DS‐II and PHQ‐9 was measured with the Cronbach's Alpha and the Omega Index coefficients^28^ using the R *userfriendlyscience* package.

#### 2.3.2 Item redundancy

Prior to estimating a network structure, redundancy between items was examined using the *node*.*redundant* function of the EGAnet package,^22^ which determines the weighted topological overlap of items and allows the combination of redundant items into distinct latent factors using the *node*.*redundant*.*combine* function. That is, items which were very strongly associated with each other and had overlapping nodes (topological overlap) in the network were combined into one composite item (or latent factor), using a mathematical model to represent their combined score as a factor score. Redundant items do not contribute to unique information in the network and can cause an overestimation of the number of dimensions in the network. Only items with both topological and conceptual overlap were combined.

#### 2.3.3 Network estimation

The network structure representing the items of the DS‐II and PHQ‐9 was estimated through exploratory graph analysis (EGA), using the EGA function^17^ of the EGAnet package. EGA uses the Gaussian Graphical Model, based on regularised partial correlations which represent the strength of association between each pair of items after associations with all other items have been controlled for. The graphical least absolute shrinkage and selection operator (GLASSO) procedure was used to select the strongest set of network connections while minimising the detection of false‐positive connections. Very small edges, likely to be due to noise, are set to zero. The network contains nodes (items) and edges (associations between items). Edges represent partial correlations between items. The network was graphically represented based on the Fruchterman–Reingold algorithm.^15^ Communities consisting of highly interconnected clusters of nodes which are only weakly connected to other nodes, were also identified by the EGA function. Communities are identified with the Walktrap community detection algorithm, which uses random walks to determine nodes clustered together.^16^ Evidence from simulation studies suggest that EGA has comparable or better accuracy in identifying the number of dimensions than factor analytic techniques.^16^

The replicability of the identified communities and the items allocated to each community was examined through a non‐parametric bootstrap procedure with 10,000 iterations, using the bootEGA function^29^ of the EGA package. This procedure estimated the proportion of times the number of communities were replicated and the proportion of times each item appeared in each community in the bootstrap replications. Higher proportions of replicability indicate more reliable and stable results. A network was also estimated in each bootstrap replication, as well as a median (or typical) network structure based on all the replicated networks. If the median network is highly similar to the originally estimated network, this indicates good replicability and stability.^29^

The centrality measure of node strength was estimated for each item. Node strength, a common and stable centrality measure, is the sum of all associations of a node with all other nodes.^18^, ^19^ The stability of the node strength index was tested by correlating the indices obtained from the full sample with the indices obtained after systematically dropping an increasing number of cases from the sample, through 2500 case‐dropping bootstrap iterations.^29^ As nodes highly correlated with other nodes can sometimes have inflated centrality,^19^ the same bootstrap procedure was repeated, but with dropping nodes, rather than cases, from the network.

## 3 RESULTS

### 3.1 Sample description

Participants without any missing items on the DS‐II or PHQ‐9 were included in this study (*n* = 1463). There were 54.7% females and 45.3% males; 76.6% were partnered; 14.2% had completed at least secondary school and 25.5% had tertiary education; the most common cancer type was breast (29.3%), followed by prostate (16.1%) and lung (11.8%); 54.9% had advanced cancer stage.

The DS‐II scores ranged from 0 to 50 with M = 15.1 and SD = 9.6. There is no standard cut‐off score for the DS‐II, but a score above the 75^th^ percentile is considered high.^25^ A DS‐II score of 21 was above the 75^th^ percentile in this study. PHQ‐9 scores ranged from 0 to 24 with M = 6.4 and SD = 4.6. Of the total sample 23.9% had PHQ‐9 score ≥10.

Crosstabulations showed that 64.9% of the sample were neither highly demoralised nor depressed; 14.2% were both demoralised and depressed; 11.3% were demoralised but not depressed; and 9.6% were depressed but not demoralised.

Table 1 show the means and standard deviations of the DS‐II and PHQ‐9 items.

**TABLE 1:** Coding and node redundancy of DS‐II and PHQ‐9 items, means and standard deviations

Scale	Item	Code in the analysis	Combined items after redundancy analysis	Range	M	SD
DS‐II	1. There is no value in what I can offer others.	worthless	DS‐II item 1 and item 13	0–4	1.63	0.96
DS‐II	2. My life seems to be pointless.	ds_pointless		0–4	0.55	0.83
DS‐II	3. My role in life has been lost	ds_role		0–4	0.51	0.90
DS‐II	4. I no longer feel emotionally in control	ds_control		0–4	0.80	0.92
DS‐II	5. No one can help me.	helpless	DS‐II item 5 and item 6	0–4	0.85	1.05
DS‐II	6. I feel that I cannot help myself	helpless	DS‐II item 5 and item 6	0–4	0.92	1.04
DS‐II	7. I feel hopeless	ds_hopeless		0–4	0.58	0.88
DS‐II	8. I feel irritable	ds_irritable		0–4	1.19	0.98
DS‐II	9. I cope fairly well with life	ds_cope		0–4	1.04	0.98
DS‐II	10. I have a lot of regret about my life	ds_regret		0–4	1.14	0.94
DS‐II	11. I tend to feel hurt easily	ds_hurt		0–4	1.33	1.00
DS‐II	12. I feel distressed about what is happening to me	ds_distress		0–4	1.67	1.09
DS‐II	13. I am not a worthwhile person	worthless	DS‐II item 1 and item 13	0–4	1.18	1.08
DS‐II	14. I would rather not be alive	death	DS‐II item 14 and PHQ−9 item 17	0–4	0.21	0.60
DS‐II	15. I feel quite isolated or alone	ds_isolated		0–4	0.63	0.93
DS‐II	16. I feel trapped by what is happening to me	ds_trapped		0–4	0.89	1.08
PHQ−9	1. Little interest or pleasure in doing things	phq_interest		0–3	0.87	0.86
PHQ−9	2. feeling down, depressed, or hopeless	phq_down		0–3	0.63	0.72
PHQ−9	3. Trouble falling or staying asleep, or sleeping too much	phq_sleep		0–3	1.33	1.06
PHQ−9	4. Feeling tired or having little energy	phq_tired		0–3	1.24	0.93
PHQ−9	5. Poor appetite or overeating	phq_appetite		0–3	0.84	0.99
PHQ−9	6. Feeling bad about yourself ‐ or that you are a failure or have let yourself or your family down	phq_failure		0–3	0.24	0.54
PHQ−9	7. Trouble concentrating on things, such as reading the newspaper or watching television	phq_concentrate		0–3	0.69	0.81
PHQ−9	8. Slowing or agitation	phq_motor		0–3	0.42	0.76
PHQ−9	9. Thoughts that you would be better off dead or of hurting yourself in some way	death	DS‐II item 14 and PHQ−9 item 17	0–3	0.15	0.41

Abbreviations: M, Mean; SD, Standard Deviation.

Positively worded DS‐II items were reverse scored.

### 3.2 Internal reliability

Both scales had high internal reliability: Omega = 0.82 with 95% CI: 0.81–0.82 for the DS‐II; and Omega = 0.90 with 95% CI: 0.89–0.90 for the PHQ‐9. Reliability for all DS‐II and PHQ‐9 items combined was also high: Omega = 0.91 with 95% CI: 0.91–0.92.

### 3.3 Item redundancy

Out of the total 25 items from the DS‐II and PHQ‐9, topological overlap between 10 pairs of items was identified. Of these, three sets of items which also overlapped conceptually were combined into the following latent factors: helplessness (‘No one can help me" and "I cannot help myself’ from the DS‐II); ideation about death and suicide (‘I would rather not be alive’ from the DS‐II and ‘Thoughts that you would be better off dead or of hurting yourself in some way’ from the PHQ‐9); and worthlessness (‘There is a lot of value in what I can offer others" and "I am a worthwhile person’). The rest of the items were not combined, as they are conceptually distinct. After the combination of redundant items, a total of 22 nodes were used for the network estimation.

### 3.4 Network estimation

#### 3.4.1 Communities

**FIGURE 1:**
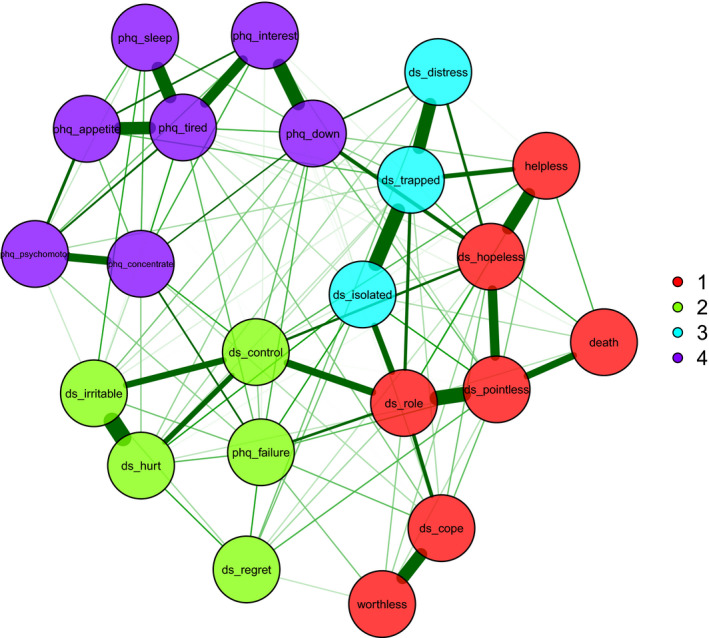
Network structure and communities of the DS‐II and PHQ‐9 items. Thickness of edge weights (green lines) reflect strength of associations between nodes

A graphical representation of the estimated network is shown in Figure 1. Four communities were identified, indicated in different node colours. We named these communities as follows: 1. Loss of hope and meaning; 2. Non‐specific emotionality; 3. Entrapment; 4. Depressive symptoms.

The first community, Loss of hope and meaning, is composed by the core demoralisation symptoms of hopelessness, helplessness, pointlessness and loss of self‐worth, as well as suicidal ideation. The second community, Non‐specific emotionality, consists of more general symptoms of distress and loss of emotional control. The third community, Entrapment, consisted of the items of isolation, feeling stuck or trapped and distress, which also had fairly strong associations with symptoms of loss of roles, helplessness and hopelessness. The fourth community, Depressive symptoms, was defined by the core depression criteria of the PHQ‐9 (including the key criteria of feeling down and loss of interest), but excluding suicidal ideation and feelings of failure. Thoughts of death and suicide had its strongest association with pointlessness, and did not show strong direct associations with the other PHQ‐9 symptoms. The PHQ‐9 feelings of failure item had reasonably strong associations with a number of PHQ‐9 and DS‐II items. Figure 1 shows that the Depressive symptoms community formed a distinctly separate cluster from demoralisation and suicidal ideation.

#### 3.4.2 Symptom associations/edges within the network

Nodes in the same community had stronger connection to each other than with nodes of other communities, as expected. Some of the edge weights indicating the strongest connections in the network were between the DS‐II symptoms of pointlessness and loss of roles, pointlessness and hopelessness, helplessness and hopelessness, isolation and entrapment; the PHQ‐9 symptoms of loss of interest and feeling down, loss of interest and tiredness, sleep problems and tiredness and appetite problems and tiredness. The edge weights (strength of associations between nodes) are shown in the Table S1.

Thoughts of death and suicide had its strongest link with pointlessness; somewhat weaker links with helplessness and hopelessness; and no noteworthy partial correlations with other PHQ‐9 symptoms. The strongest connections between DS‐II and PHQ‐9 symptoms were between hopelessness (DS‐II) and feeling down (PHQ‐9), and loss of roles (DS‐II) and feelings of failure (PHQ‐9). Thus thoughts of death and suicide were indirectly linked to DSM‐5 depression though hopelessness and feeling down.

#### 3.4.3 Stability of network estimates

The stability of the estimated network was assessed though bootstrap replications and the following indicators of stability were examined: (a) number of communities; (b) structural consistency of each community; (c) frequency of item replications across each communities; and standardised node loads for each community.

##### (a) Number of communities

In the bootstrap replications four communities were derived in the network in 82.4% of the replications, suggesting the number of communities are stable and replicable (Median of derived communities = 4, 95% CI = 3.20 to 4.80).

##### (b) Structural consistency of the communities

Structural consistency is the proportion of times the item composition of each community is replicated in the bootstrap. Values range from 0 to 1. The following structural consistencies were obtained: Community 1 (Loss of hope and meaning) 0.50; Community 2 (Non‐specific emotionality) 0.34; Community 3 (Entrapment) 0.62; Community 4 (Depressive symptoms) 0.99.

##### (c) Item stability

**FIGURE 2:**
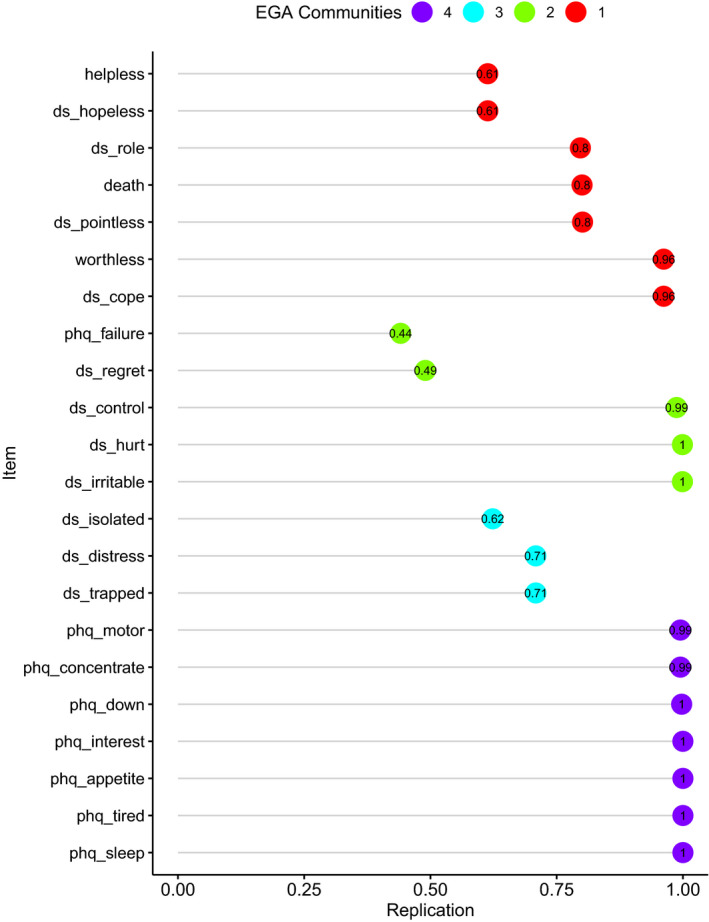
Probability of each symptom belonging to the community it was originally identified in by EGA (results from bootstrap with 10,000 iterations)

Item stability is the proportion of times each item is replicated in each community by the bootstrap. Figure 2 shows the proportion of times each item is replicated in its original community specified by the EGA network. The proportion of times each item was replicated in each community is further shown in Table 2.

**TABLE 2:** Frequencies of symptom replication in each community (results from bootstrap with 10,000 iterations)

Item	1 (Loss of hope and meaning)	2 (Non‐specific‐emotionality)	3 (Entrapment)	4 (Depressive symptoms)	5
helpless (latent factor)	**0.613**		0.381		0.005
ds_hopeless	**0.614**		0.381		0.005
ds_role	**0.796**	0.004	0.188		0.011
death (latent factor)	**0.800**		0.191		0.008
ds_pointless	**0.801**		0.190		0.008
worthless (latent factor)	**0.818**	0.001	0.004	0.002	0.025
ds_cope	**0.818**	0.001	0.004	0.002	0.025
phq_failure	0.243	**0.375**	0.032	0.176	0.024
ds_regret	0.273	**0.457**	0.175	0.015	0.012
ds_control	0.006	**0.988**	0.003		0.003
ds_hurt		**0.999**			
ds_irritable		**0.999**			
ds_isolated	0.370	0.004	**0.623**		0.003
ds_distress	0.288	0.003	**0.709**		
ds_trapped	0.288	0.003	**0.709**		
phq_motor		0.004		**0.994**	
phq_concentrate		0.004		**0.994**	
phq_down			0.002	**0.998**	
phq_interest				**1.000**	
phq_appetite				**1.000**	
phq_tired				**1.000**	
phq_sleep				**1.000**	

Values ≥ 0.300 are in bold.

Figure 2 and Table 2 show that most items were replicated in their original community in a very high proportion of the replications. However, there were some items which were also replicated in other communities with fairly high proportions. Feelings of failure (PHQ‐9) and regret (DS‐II) were only replicated in Community 2 (Non‐specific emotionality) in 0.38 and 0.46 of the replications, which could explain the lower structural consistency of this community. Community 4 (Depressive symptoms) was the most stable with all its items having nearly zero probability of being replicated in other communities. The other three communities comprising mainly the DS‐II items and the combined ideation of death and suicide items, were less stable and more interconnected with each other, but clearly distinct from Community 4 (Depressive symptoms).

##### (d) Standardised node loadings

The standardised node strengths, or loadings of each symptom on the network communities, (these are based on partial correlations and are interpreted in a similar way to factor analysis loadings) are shown in the Table S2. More central items tend to crossload on more than one community.

The median network derived from the 10,000 bootstrap iterations was very similar to the original network in Figure 1. This provides further evidence for the good stability of the network estimates.

### 3.5 Centrality

**FIGURE 3:**
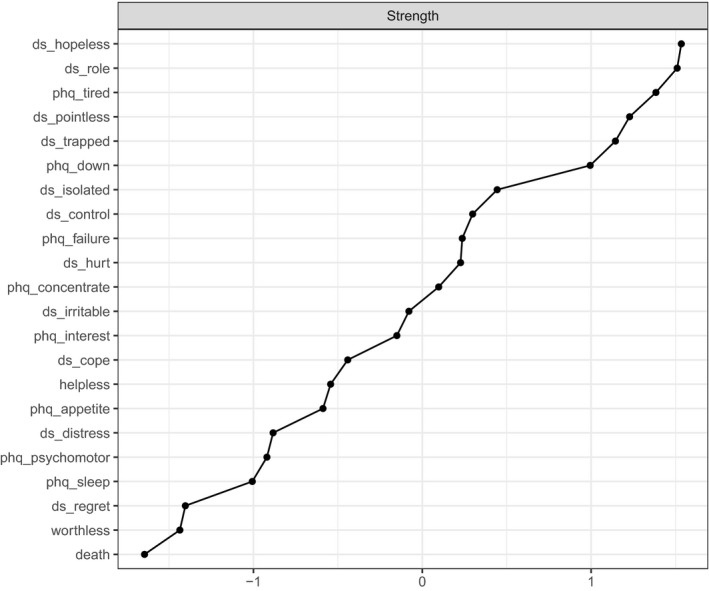
Node strength centrality, where its strength centrality results from the sum of all associations of a node with all other nodes

The node strength centrality indices in Figure 3 show that the most central nodes in the network were hopelessness, loss of role, tiredness (PHQ‐9), pointlessness, entrapment, hopelessness and isolation. The least central were ideation of death and suicidal thinking, worthlessness, regret, sleep problems (PHQ‐9), psychomotor symptoms (PHQ‐9), distress, appetite problems (PHQ‐9) and helplessness.

### 3.6 Stability of the centrality indices

The stability of node strength was good. The bootstrap procedure with 2500 iterations showed that 75% of the individual cases in the sample could be dropped, maintaining a correlation of 0.90 between the new values of node strength with those of the original sample (shown in Data S3). When nodes were dropped from the bootstrap, rather than cases, the node strength remained reasonably stable for each node until 40% to 50% of nodes were dropped (Data S4).

## 4 DISCUSSION

### 4.1 Communities of symptoms of demoralisation and depression

We used a sample of 1527 German cancer patients to investigate the inter‐relationships between symptoms of depression and demoralisation. Exploratory graph analysis identified four communities: 1. Loss of hope and meaning; 2. Non‐specific emotionality; 3. Entrapment; 4. Depressive symptoms. Depressive symptoms, except for suicide ideation and fear of failure, clustered in a distinct and stable community clearly separated from demoralisation. Suicidal ideation and thoughts of death were more closely related to demoralisation than to the other depressive symptoms, the strongest link being with pointlessness. Fear of failure was linked to both depressive and demoralisation symptoms. There was some overlap between the three demoralisation communities. This is consistent with the DS‐II validation study,^25^ which showed that the DS‐II can be used as a unidimensional tool.

Our results are overall similar to an earlier Italian study with 447 medical inpatients,^30^ which also used EGA to examine the inter‐relationship between depression and demoralisation symptoms. Belvederi Murri et al. used the original longer 24‐item version of the Demoralisation Scale. They also identified four communities. A community consisting of anhedonia and neurovegetative depression symptoms emerged, which was distinctly separate from demoralisation. Suicidal ideation and thoughts of death were part of a community consisting of core demoralisation symptoms, such as loss of purpose and hopelessness. The PHQ‐9 symptoms of feelings of failure and low mood had stronger links with demoralisation than depression. Pointlessness and hopelessness were among the most central symptoms, similar to our study. Although the results of our study and that of Belvederi Murri et al. point to similar overall conclusions, there were also some differences in how individual items loaded on the three demoralisation communities. Our Loss of hope and meaning community was similar to Belvederi Murri et al.'s lack of purpose community. Belvederi Murri et al.'s loss of self‐worth and frustrated isolation were somewhat different in item content from our demoralisation community. These differences may be cultural and due to the use of the longer scale. Our study was based on a much larger sample in a different cultural setting. We used the briefer DS‐II which is well validated and more parsimonious in evaluating demoralisation symptoms. The demoralisation communities tend to be less stable and generally vary somewhat from study to study. However, overall similarities across the two studies are greater than the differences.

### 4.2 Link with adjustment disorder

The results of the present study are consistent with a previous study^8^ which identified a class reflecting poor coping without anhedonia and other classic depression symptoms, but linked to suicidal ideation. The present study is also consistent with studies showing that suicidality has stronger links with demoralisation than depression in cancer patients.^4^, ^5^, ^6^, ^7^ For people facing the existential threat of cancer, demoralisation symptoms with suicidal ideation, but without anhedonia, may indicate poor psychological adjustment to the stressors of their illness. Thus, demoralisation may be an important element of AD which contributes importantly to the independent link between AD and suicidality.^11^, ^12^

Criticism exists about the diagnostic criteria for adjustment disorder being too subjective and non‐specific.^12^ The phenomenology of demoralisation offers a stronger set of diagnostic criteria to enrich the diagnosis of adjustment disorder as a full syndrome disorder rather than a subthreshold one. Hopelessness, pointlessness, entrapment and the loss of roles that deliver purpose and meaning to life constitute a clear cluster of symptoms associated with the loss of morale that is central to adjustment disorder. These reinforce the recent latent class structure that positioned adjustment disorder separately to depressive‐anxiety disorders.^8^

### 4.3 Clinical implications: intervention targets

Exploratory graph analysis provides an understanding of which symptoms are centrally located in the hub of a community of symptoms. These central symptoms could become appropriate intervention targets to begin to ameliorate each cluster of symptoms.

Entrapment is one such example of a core symptom, which is strongly associated with both the development of deep distress and a feeling of social isolation. The stressful predicament can appear to leave a patient stuck, feeling unable to control such circumstances and caught in a helpless position. Feeling trapped in this manner induces a powerful sense of defeat, which can be generalised to loss of all control. The clinician can cultivate realisation that aspects of life can be brought under control, and acceptance that not all of life needs to be controlled. For the medically ill, optimal symptom control can improve quality of life, while reconnection with friends is established. As some mastery is achieved, the initial sense of feeling trapped is lessened, distress reduced and a greater connection with others achieved through targeting entrapment as a core symptom to focus on.

Similarly, hopelessness, pointlessness and loss of roles sit centrally as set of core symptoms that generate the state of demoralization, including its propensity to induce suicidal thinking. Restaging an illness such as cancer with the outcome of a poorer prognosis can cause loss of hope. When a particular hope is reduced––here, for instance, the hope for a long life––the clinician needs to refocus from this particular hope by drawing upon the more generalized hope involved with quality of life, and concentrate on worthwhile here and now experiences. Likewise, when the patient feels that the value and point of life is damaged by their illness, the clinician identifies continuing sources of meaning and fulfilment, sometimes termed generalised meaning, to counter any apparent pointlessness. Furthermore, the many available roles that a person has in life can be brought into focus when hopelessness and pointlessness become problematic. Relationally, there is often a role as spouse, parent or grandparent that has drifted out of focus; or there may be creative roles as an artist, a biographer leaving their narrative as a legacy, or some craft that produces a gift for another. Rather than allow the sick role to dominate, the clinician gently guides the patient into an appreciation that continuing roles persist that can deliver joy and self‐worth.

A deeper clinical exploration of the phenomenology of demoralization, with its low morale and poor coping, provides the clinician with potent and constructive targets to address in restoring well‐being and fostering reconnection with the innate resilience of the person. Not only is the process of adjustment considered as a primary concern for the patient, but also great utility lies in the intervention options that open up as a result of this richer conceptualisation.

### 4.4 Strengths and limitations

A limitation of this study is its combination of three samples obtained with varied recruitment strategies. However, the ultimately large sample incorporating different treatment settings and capturing a range of tumour sites is a strength. The 35% prevalence of high levels of demoralisation and/or depression in this sample is representative of cancer patient populations.^1^ However, for further validation of the results the study needs to be replicated in different populations, including among people with cancer or other physical illnesses who fulfil DSM‐5 diagnostic criteria for mental disorders. The study is cross‐sectional and cannot determine the directionality of the network edges or the causality of central symptoms.

## CONFLICT OF INTEREST

The authors declare no conflict of interest.

## AUTHOR CONTRIBUTIONS

IB and DWK: conception, writing – original draft; IB: formal analysis; AM and SV: provided the data from a study they had previously conducted of 1529 German cancer patients and edited the manuscript, writing – review and editing; MBM and LG: writing – review and editing.

## ETHICAL APPROVAL

Approval was obtained from the Hamburg Medical Association Ethics Committee.

## Supporting information

Supplementary Material

## Data Availability

Shared data are not available.
