# Supplementary material for: Demoralisation and its link with depression, psychological adjustment and suicidality among cancer patients: A network psychometrics approach
Source: Cancer Med. 2021 Dec 4;11(3):815–25. doi: 10.1002/cam4.4406 (PMC8817077; doi:10.1002/cam4.4406)
Supplement: Supplementary file 1 — Supplementary Material [file CAM4-11-815-s001.pdf]

Supplementary materials

S1. Network edge weights (strength of associations between nodes).

	ds_pointless	ds_role	ds_control	helpless	ds_hopeless	ds_irritable	ds_cope	ds_regret	ds_hurt	ds_distress	worthless	death	ds_isolated	ds_trapped	phq_interest	phq_down	phq_sleep	phq_tired	phq_appetite	phq_failure	phq_concentrate	phq_motor
**ds_pointless**	1																					
**ds_role**	0.31	1																				
**ds_control**	-	0.19	1																			
**helpless**	0.05	0.04	0.07	1																		
**ds_hopeless**	0.22	0.09	0.11	0.27	1																	
**ds_irritable**	-	-	0.17	-	-	1																
**ds_cope**	0.06	0.13	-	-	0.05	-	1															
**ds_regret**	0.07	0.03	-	-	0.03	0.04	-	1														
**ds_hurt**	0.02	-	0.16	-	0.01	0.34	-	0.08	1													
**ds_distress**	-	-	-	-	0.12	0.02	-	0.05	0.04	1												
**worthless**	0.03	0.04	-	-	0.04	-	0.27	0.02	-	-	1											
**death**	0.18	0.03	-	0.07	0.08	-	-	-	-	-	-	1										
**ds_isolated**	0.10	0.17	0.02	0.01	0.03	-	0.04	0.05	0.09	-	-	0.03	1									
**ds_trapped**	-	0.12	0.02	0.15	0.08	0.01	0.03	-	0.02	0.28	-	-	0.30	1								
**phq_interest**	0.03	-	0.00	-	0.01	-	-	-	-	-	-	-	0.01	-	1							
**phq_down**	0.06	-	0.06	0.04	0.13	0.05	-	-	0.04	0.10	-	-	0.02	0.05	0.29	1						
**phq_sleep**	-	-	-	-	-	0.08	-	-	-	-	-	-	-	-	-	0.05	1					
**phq_tired**	-	-	-	-	0.02	0.01	0.05	-	-	-	-	0.01	0.03	0.01	0.24	0.07	0.28	1				
**phq_appetite**	-	-	-	-	-		0.00	-	-	-	-	-	-	0.06	0.10	-	0.07	0.27	1			
**phq_failure**	0.07	0.11	0.04	-	-	0.05	0.06	0.09	0.07	-	0.05	-	0.09	-	0.01	0.07	-	0.06	-	1		
**phq_concentrate**	-	-	0.08	-	-	0.03	0.03	-	0.04	-	-	-	-	-	0.07	0.10	0.07	0.09	0.06	0.10	1	
**phq_motor**	-	-	-	-	-	0.02	-	-	-	-	-	-	-	0.03	0.06	-	0.02	0.10	0.12	0.04	0.20	1

Note: As these are based on partial correlations, loadings ≥ 0.2 indicate a medium association and ≥ 0.3 strong association.

S2. Node loadings for each community based on partial correlations.

	1	2	3	4
**ds_pointless**	**0.346**	0.08	0.057	0.036
**ds_role**	**0.261**	0.169	0.175	
**helpless**	**0.176**	0.034	0.095	0.018
**ds_hopeless**	**0.304**	0.071	0.136	0.066
**ds_cope**	**0.211**	0.030	0.042	0.035
**worthless**	**0.156**	0.037		
**death**	**0.146**		0.019	0.004
**ds_control**	0.151	**0.187**	0.025	0.060
**ds_irritable**		**0.302**	0.022	0.076
**ds_regret**	0.062	**0.102**	0.065	
**ds_hurt**	0.013	**0.323**	0.092	0.034
**phq_failure**	0.118	**0.123**	0.055	0.118
**ds_distress**	0.048	0.057	**0.166**	0.042
**ds_isolated**	0.151	0.126	**0.181**	0.020
**ds_trapped**	0.154	0.030	**0.346**	0.064
**phq_interest**	0.017	0.004	0.004	**0.310**
**phq_down**	0.096	0.110	0.099	**0.209**
**phq_sleep**		0.043	0	**0.201**
**phq_tired**	0.031	0.034	0.022	**0.433**
**phq_appetite**			0.039	**0.257**
**phq_concentrate**	0.013	0.126	0	**0.245**
**phq_motor**		0.031	0.02	**0.207**

Note: The node loadings reflect each nodes contribution to the coherence of the communities. They are interpreted in a similar way to exploratory factor analysis loadings, but as they are based on partial correlations, loadings ≥ 0.2 indicate a medium association and ≥ 0.3 strong association.

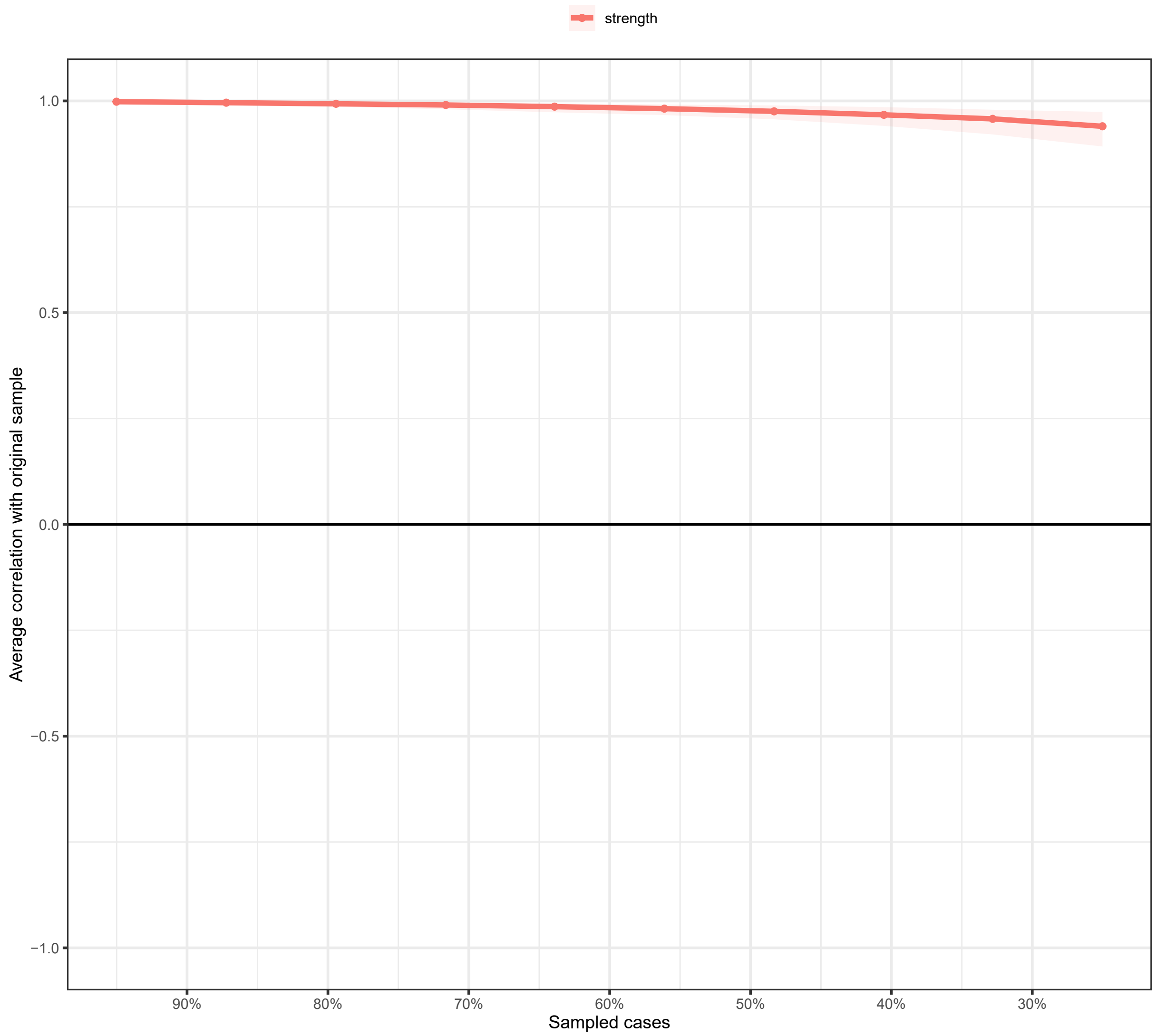

S3. Stability of node strength centrality estimated with 2,500 case dropping bootstrap iterations.

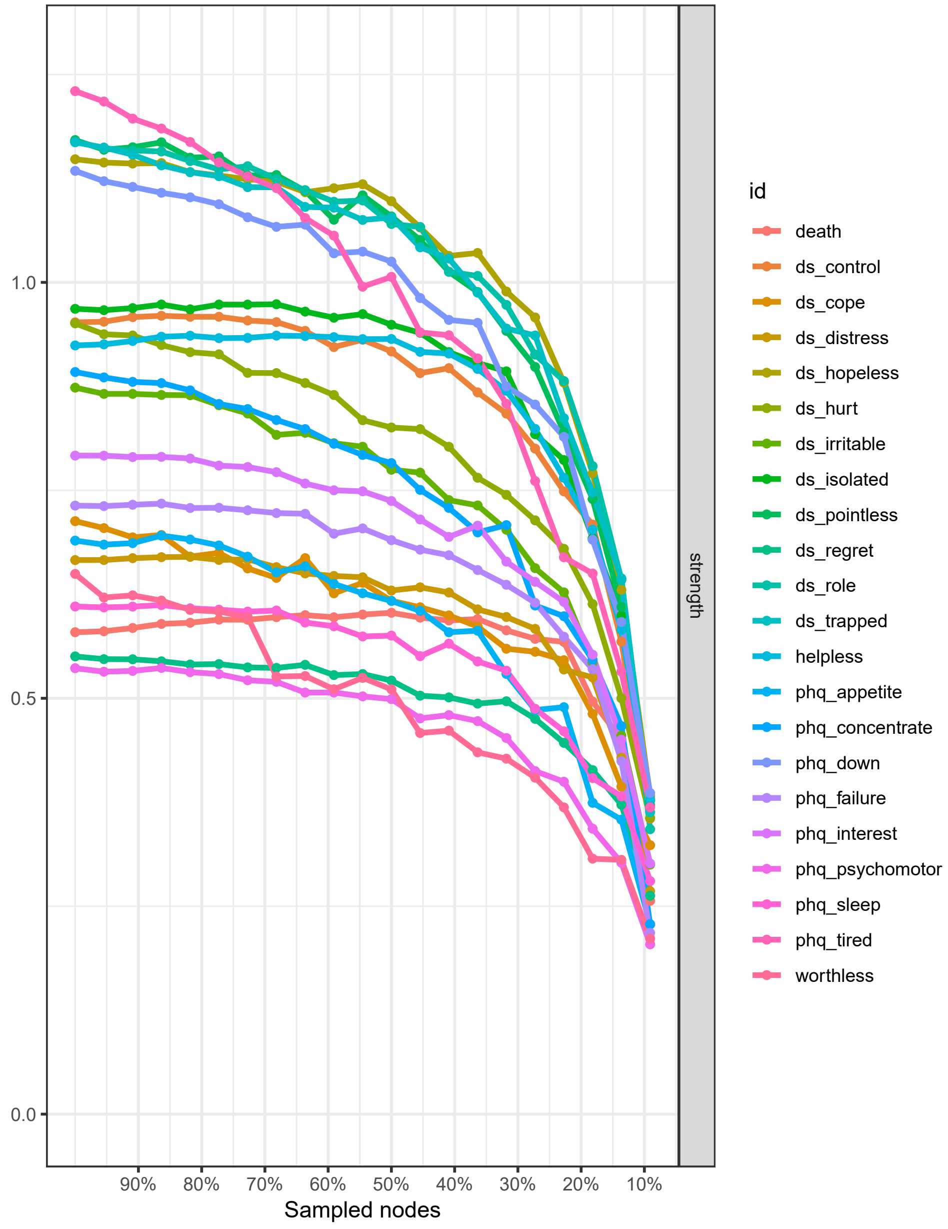

S4. Stability of node strength centrality for each node estimated with 2,500 node dropping bootstrap iterations. The X-axis shows the % of nodes (items) sampled. Parallel item lines imply a large stability of the centrality estimates.
